# Supplementary material for: H∞-based control of multi-agent systems: Time-delayed signals, unknown leader states and switching graph topologies
Source: PLoS One. 2022 Apr 28;17(4):e0263017. doi: 10.1371/journal.pone.0263017 (PMC9049309; doi:10.1371/journal.pone.0263017)
Supplement: Attachment — Submitted filename: Responses_PONE-D-21-35036.pdf [file pone.0263017.s001.pdf]

Original Manuscript ID: PONE-D-21-35036

Original Article Title: "H∞-based control of multi-agent systems: Time-delayed signals, unknown leader states and switching graph topologies"

Dear Reviewer,

We sincerely thank you, for the time spent and the effort made in the reviewing process. Following the provided insightful comments and valuable suggestions, we have further revised the paper. All changes has been highlighted by yellow color.

Best Regards

The authors

Additional Editor Comments:

Two reviews have been collected. Both reviewers agree that the paper contains publishable results, but they also raised critical comments. Some of them are summarized below.

1) Literature review is not sufficient.
2) The motivation of this work is unclear. For instance, why local stability is studied instead of global stability?
3) Practical examples need to be presented.

4) Math symbols are not defined.

5) Discussions/clarifications of the results need to be given

5. Review Comments to the Author

Please use the space provided to explain your answers to the questions above. You may also include additional comments for the author, including concerns about dual publication, research ethics, or publication ethics. (Please upload your review as an attachment if it exceeds 20,000 characters)

Response: Thank you very much for your comments. All comments have been carefully investigated and addressed in below.

Reviewer #1:

The authors in this paper developed H_∞-based leader-following scheme for multi-agent systems. However, I have some remarks that if they are answered, it can improve the manuscript:

Reviewer#1, Comment#1: In the section "Simulations' authors chosen some parameters for simulation, but absent any information of justification of chosen parameters. Why the time-delay was selected as 200(ms)? The parameters must be related with any physical model.

Response: Thank you for your attention. Indeed, the value of time-delay for the numerical example is arranged due to the feasibility of LMIs. For the practical example, the value of time-delay is adjusted based on the physical structure of the model. It should be noted that for a given time-delay the free parameters are obtained such that the LMIs to be satisfied. The following statements added:

> The value of time-delay this example is arranged due to the feasibility of LMIs. For the practical example, the value of time-delay is adjusted based on the physical structure of the model. It should be noted that for a given time-delay the free parameters are obtained such that the LMIs to be satisfied.

Reviewer#1, Comment#2: Must be added expended discussion and comments about figures 5-15.

Response: Thank you for your valuable comment. This matter has been modified for the revised version. The following Remark has been added:

> **Remark 5.** The results in Fig.3- 16, show that the suggested controller well handle the

time-varying gain perturbations, and the designed observer well estimates the leader's states. In addition to perturbations, it is seen that the effect of time-delay and switching topology is well tackled. Furthermore, the effect of external disturbances are handled by the designed H_∞-based scheme. It is demonstrated that the suggested consensus scenario, is good effective in the presence of external disturbance and other perturbations such as time-delay and switching tocology. A good and desired convergence to zero level is seen in the error trajectories.

Reviewer#1, Comment#3: conclusion authors write about stability of the suggested distributed observer and controller, but in the paper are absent any estimation of criteria stability.

Response: Thank you for bringing this into our attention. Indeed, the distributed observer has been designed to estimate the leader's states and based on the estimation, the distributed controller has been applied to the system to solve the consensus problem. Therefore, the role of observer with stability analysis of the estimation error (error between the states of the leader and its estimations) has been analyzed. To this end, Lyapunov-Krasovskii functional approach has been considered and it is guaranteed that the error converges to the origin. For further clarification and better addressing your valuable comment, the following Remark has been added:

Remark 6. It should be noted that the distributed observer is designed to estimate the leader's states, and based on the estimation, the distributed controller is applied to the system to solve the consensus problem. Therefore, the role of observer with stability analysis of the estimation error (error between the states of the leader and its estimations) is analyzed. To this end, Lyapunov-Krasovskii functional approach is considered and it is guaranteed that the error converges to the origin.

Reviewer#2

Reviewer#2, Comment#1:. The abstract and conclusion can be enhanced by capturing the main results as well as the significance.

Response: Thank you for your comment. This matter has been modified in the revised version.

Abstract

The paper investigates leader-following scheme for nonlinear multi-agent systems (MASs). The network of agents involves time-delay, unknown leader's states, external perturbations, and switching graph topologies. Two distributed protocols including a consensus protocol and an observer are utilized to reconstruct the unavailable states of leader in a network of agents. The H_∞-based stability conditions for estimation and consensus problems are obtained in the framework of linear-matrix inequalities (LMIs) and Lyapunov-Krasovskii approach. It is ensured that each agent achieves the leader-following agreement asymptotically. Moreover, the robustness of the control policy concerning a gain perturbation is guaranteed. Simulation results are performed to assess the suggested schemes. It is shown that the suggested approach gives a remarkable accuracy in the consensus problem and leader's states estimation in the presence of time-varying gain perturbations, time-delay, switching topology and disturbances. The H_∞ and LMIs conditions are well satisfied and the error trajectories are well converged to origin.

Reviewer#2, Comment#2: Survey of existing literature is not sufficient. It would useful to include in the Introduction of the paper some discussion on other possible real applications of the obtained results.

Response: Thank you for your comment. Some more discussion added.

adaptive distributed scheme has been designed in [8] for the LF consensus problem. Consensus tracking problem of stochastic nonlinear MASs has been investigated by utilizing event-triggered mechanisms in [9]. Recently, LF consensus of MASs with semi-Markov jump parameters has been analyzed by implementing hybrid event-triggered strategy to tackle the transmission frequency of surplus data packets [10]. The LF problem of stochastic MASs subject to multiplicative noises has been studied via output feedback control policies in [11]. The consensus tracking problem of MASs in the presence of unknown dynamics/nonparametric uncertainties has been studied through designing a distributed control law in [12]. Based on an impulsive model, the fixed-time tracking control problem has been studied for a set of planar agents in a surveillance network in [13].

in second-order dimensions under STs has been studied in the literature [20, 21]. Distributed control policies have been proposed for mobile autonomous agents with leaders under switching directed network topologies in [22]. Considering the static positions for leaders and an undirected switching graph topology in [23], the convergence problem of followers to the convex hull has been studied. Based on a distributed control scheme, the LF consensus of MASs with switching topologies and stochastic disturbances has been analyzed in [24]. Utilizing an average dwell time condition and distributed control policies, the LF consensus problem of MASs with unknown control/output directions and switching topologies has been studied in [25]. For MASs with one-sided Lipschitz nonlinear dynamics, the LF consensus problem has been studied under switching topologies in [26]. Moreover, the LF consensus problem for MASs has been analyzed by designing an event-triggered control scheme in [27].

Reviewer#2, Comment#3:.. The authors have to state the weakness in previous works which motivated this study.

Response: Thank you for your attention. The main contribution of the paper in comparison with the previous studies are summarized as follows:

- In comparison with the LF consensus problem analysis or tracking problem investigation where the leader's or target's states are accessible, in this paper this assumption is violated and they are completely inaccessible; hence, an observer in distributed form is designed to reconstruct the leader states. Moreover, the network of the agents is subjected to time-delay.
- The switching topologies are considered for the communication network. In addition, the influence of delay in the states of the leader and each follower is investigated in this paper. Furthermore, due to the actuator degradations, time-varying gain perturbation is considered and robustness of the distributed controller for gain perturbations is studied.
- H_∞ LF consensus problem of the time-delay nonlinear MAS with unknown leader's states is investigated based on a prescribed H_∞ disturbance attenuation along with a DSO and a distributed controller.

Reviewer#2, Comment#4:. Some abbreviations need to be extended when mentioned for the first time, like LMASs, NMASs, etc.

Response: Thank you for your comment. This matter has been corrected in the revised version of the manuscript.

Reviewer#2, Comment#5:. The local asymptotic stability is goal of this study. Why the authors did not address the global asymptotic stability.

Response: Thank you for your comment. In two Theorems we prove the asymptotic stability not global stability. We prove that by the designed scheme, the followers asymptotically observe the leader, and the H∞ leader following consensus is satisfied. To better clarify and address your valuable comment, the following Remark added:

Remark 1. In two Theorems, the asymptotic stability is proved. It is shown that by the designed scheme, the followers asymptotically observe the leader, and the H_∞-based leader following consensus is satisfied.

Reviewer#2, Comment#6:. The design analysis is very good, but the choice of design parameters is still a challenging problem. I suggest to perform one of the modern techniques in tuning these design parameters.

Response: Thank you for your comment. We are going to apply your valuable comment in our future studies. To give a direction to the readers we have added the following statements to the conclusion:

results. It should be noted that the choice of design parameters is still a challenging problem. I future studies, the modern techniques such as neuro-fuzzy approaches can be developed for better tuning of these design parameters.

Reviewer#2, Comment#7:. The author needs to give some practical examples to support the work.

Response: Thank you for your comment. Actually we are going to practically apply the suggested approach on searching robotic application. Because of Covid19 condition, our access to the Lab has been limited. Then we hope that the respectful reviewer give us an opportunity to address this comment in our future paper. We have added the following Remark:

Remark 7. It should be noted that, the most important practical restrictions such as time-delay, external disturbances, time-varying gain perturbations and switching topologies, have been considered in the control design. Then, the suggested approach can be easily used in real-world applications. For our future studies, the designed approach will be applied for a group of searching robots.

Reviewer#2, Comment#8:. This study has to conduct a comparison in performance with other strategies.

Response: Thank you for your comment. This issue has been investigated in the revised version.

To better show the leader's states estimation accuracy and also consensus performance, a comparison with similar approaches are provided. The values of root-mean-square-errors for SMC [41], L2-L_∞ [42] and proposed approach are given in Table 1. The results of Table 1, clearly show the superiority of the designed approach.

Table 1. Comparisons of RMSEs.

Error	Proposed	SMC [41]	L2-L_∞ [42]
ε_{11}	0.4748	0.8701	0.4801
ε_{12}	0.3075	0.5407	0.4504
ε_{13}	0.2250	0.2147	0.6327
ε_{14}	0.4133	0.7414	0.5447
ε_{15}	0.4261	0.5784	0.6014

Reviewer#2, Comment#9:. Many symbols of equations are not defined. See Eq.(6)-Eq.(11).

Response: Thank you for your attention. The paper thoroughly rechecked for missed definitions.

Reviewer#2, Comment#10:. The switching from Eq.(20) to Eq.(21) is not clear.

Response: Thank you for your comment. Indeed, in Eq. (20), we have an unknown parameter due to the existence of the parametric uncertain matrix (norm-bounded uncertainty in the gain of controller).

To tackle this issue and achieve an upper bound, and convert the problem and compute the gains in terms of LMIs for the stability analysis, we use Lemma 2. Moreover, by defining K and phi in the form of the paper, one can acquire the gain of controller and observer simultaneously and from solving the LMI of Theorem 1.

The following statements added for more clarifications:

> In (20), we have an unknown parameter due to the existence of the parametric uncertain matrix (norm-bounded uncertainty in the gain of controller). To tackle this issue and achieve an upper bound, and convert the problem and compute the gains in terms of LMIs for the stability analysis, we use Lemma 2. Moreover, by defining $\varphi = B^T P$ and $K = B^T Q$, one can acquire:

Reviewer#2, Comment#11:. The authors have to explain the derivation of Eq.(22).

Response: Thank you for your attention. Eq. (22) is achieved based on the Eq. (21). To this goal, based on the definitions of the Kronecker product, and definition of Lambda and mu which are proposed in the Theorem 1, one can acquire the upper bound based on the dynamics of consensus and estimation errors.

> Therefore, considering (21), based on the definitions of the Kronecker product, and definition of λ and μ which are proposed in the Theorem 1, one can acquire the upper bound based on the dynamics of consensus and estimation errors. Then, one can infer that

Reviewer#2, Comment#12:. The authors has to explain how the alpha's and beta's parameters have been chosen.

Response: Thank you for your comment. Indeed, beta is related to the cost function (26) and we try to reduce it in order to reduce the effect of external disturbances on the consensus error dynamics. Moreover, there is no restriction in the choice of alpha's, since they can enable us to tackle the feasibility of the problem/LMIs. The following Remark added:

> **Remark 4.** The parameter β is related to the cost function (26) and we try to reduce it in order to reduce the effect of external disturbances on the consensus error dynamics. Moreover, there is no restriction in the choice of α, since they can enable us to tackle the feasibility of the problem/LMIs.

Reviewer#2, Comment#13:. The discussion of results needs some clarification.

Response: Thank you for your comment. Some more statements added about the results:

> **Remark 5.** The results in Fig.3- 16, show that the suggested controller well handle the time-varying gain perturbations, and the designed observer well estimates the leader's states. In addition to perturbations, it is seen that the effect of time-delay and switching topology is well tackled. Furthermore, the effect of external disturbances are handled by the designed H_∞-based scheme. It is demonstrated that the suggested consensus scenario, is good effective in the presence of external disturbance and other perturbations such as time-delay and switching tocology. A good and desired convergence to zero level is seen in the error trajectories.

Reviewer#2, Comment#14:. The conclusion has to be extended with future work.

Response: Thank you for your valuable comment. This matter has been modified for the revised version.

> results. It should be noted that the choice of design parameters is still a challenging problem. I future studies, the modern techniques such as neuro-fuzzy approaches can be developed for better tuning of these design parameters.
